# Novel Extranasal Tear Stimulation: Pivotal Study Results

**DOI:** 10.1167/tvst.9.12.23

**Published:** 2020-11-17

**Authors:** Marco H. Ji, Darius M. Moshfeghi, Laura Periman, David Kading, Cynthia Matossian, Gerald Walman, Scott Markham, Andy Mu, Ann Jayaram, Michael Gertner, Paul Karpecki, Neil J. Friedman

**Affiliations:** 1 Horngren Family Vitreoretinal Center, Byers Eye Institute, Department of Ophthalmology, Stanford University School of Medicine, Palo Alto, CA, USA; 2 Department of Medicine, Icahn School of Medicine at Mount Sinai, New York, NY, USA; 3 Evergreen Eye Center, Seattle, WA, USA; 4 Specialty Eyecare Group, Seattle, WA, USA; 5 Matossian Eye Associates, Pennington, NJ, USA; 6 Arizona Eye Specialists, Phoenix, AZ, USA; 7 M&M Eye Institute, Prescott, AZ, USA; 8 NV Eye Surgery, Las Vegas, NV, USA; 9 Mid-Peninsula Ophthalmology Medical Group, Menlo Park, CA, USA; 10 Olympic Ophthalmics, Inc., Issaquah, WA, USA; 11 Kentucky Eye Institute, Lexington, KY, USA

**Keywords:** dry eyes, dry eye disease, neurostimulation, lacrimal gland

## Abstract

**Purpose:**

To evaluate the efficacy and safety of iTEAR, a novel, portable, sonic external neuromodulation device, for the treatment of dry eye disease (DED).

**Methods:**

This was a multicenter, open-label, single-arm clinical trial that included adult patients with DED with a Schirmer score of ≤10 mm in at least one eye. Enrolled subjects were instructed to apply the study device at least twice per day for 30 seconds bilaterally to the external nasal nerve. After the initial baseline visit, patients were followed up at days 3, 14, 30, 90, and 180. The primary efficacy endpoint was the Schirmer index (change from unstimulated to stimulated tear production as measured by the Schirmer test) at day 30. The major secondary endpoint was the change in symptoms of DED at day 30 evaluated using the Ocular Surface Disease Index (OSDI).

**Results:**

A total of 101 subjects evaluated at day 30 had a mean Schirmer index of 9.4 mm (95% confidence interval [CI], 7.4–11.3), and the baseline OSDI improved by an average of 14.4 (95% CI, 11.1–17.7). Both endpoints were highly statistically and clinically significant at all time points. There were two mild unanticipated adverse events definitely related to the device.

**Conclusions:**

The safety and efficacy of the iTEAR device observed in this study support its indication for treating DED.

**Translational Relevance:**

Neurostimulation has the potential to improve signs and symptoms of DED.

## Introduction

Dry eye disease (DED) represents a spectrum of multifactorial ocular diseases characterized by abnormal quality and/or quantity of the tear film, resulting in the loss of homeostasis of the lacrimal functional unit (LFU) due to impairment of one or more of its components. The prevalence in the United States of moderate to severe DED has been estimated to be 3.2 million women and 1.7 million men over 50 years old.^1^^,^^2^ DED is usually accompanied by inflammation and an increase in lacrimal osmolarity.^3^^,^^4^ The LFU is a complex system comprised not only of lacrimal film but also the entire ocular surface (cornea and conjunctiva), as well as lacrimal glands, meibomian glands, goblet cells, and an intricate network of afferent and efferent neurons. In addition to discomfort and decreased quality of life, tear film instability can cause dynamic higher order aberrations and refractive micro-fluctuations between blinks, with compensatory accommodative corrections leading to eyestrain as well as fluctuations in visual acuity.^5^

Symptomatic treatment using artificial tears remains the mainstay for DED management. In patients with specifically identified etiologies, immunosuppressive drugs, tear retention using punctal plugs, and various approaches aimed at improving meibomian gland function have all been employed with variable success. Neurostimulation of the LFU is an approach grounded in well-established physiologic principles; for example, inactivation of the nasolacrimal pathway through nasal anesthesia has been shown to decrease basal tear production by 34%.^6^ It has been known for many decades that tears can be induced through stimulation of the nasal mucosa; recently, it has been shown that electrical stimulation of the nasal mucosa results in activation of the anterior ethmoidal branch of the trigeminal nerve and stimulation of the LFU.^7^ The concept of electrical neurostimulation of the LFU has now been embodied in a device approved recently by the Food and Drug Administration (FDA) and validated through pivotal trials (TrueTear, Allergan plc, Dublin, Ireland).^8^

Olympic Ophthalmics, Inc. (Issaquah, WA) has developed a novel, portable, sonic external neurostimulation device, the iTEAR, which introduces two variations on stimulation of the LFU: (1) external application, and (2) external nasal nerve stimulation. In this study, we aimed to evaluate the efficacy, safety, and usability of the iTEAR device, which has recently been approved for marketing by the FDA and will be commercialized under the name iTEAR100.

## Methods

This study was a multicenter, prospective, open-label clinical trial to assess the safety, tolerability, and effectiveness of the iTEAR device in subjects with DED over 30 days. Sites were chosen to obtain a diverse mix of subjects and eye care providers. The primary endpoint was at 30 days; however, subjects were given the option to continue to 180 days to obtain additional safety and efficacy data. Subjects were permitted to continue with their medication regimens throughout the study but were asked not to change medications over the first 30 days. Investigators were asked to recruit subjects who were on stable medication regimens for DED. This design was chosen to ensure that a diverse group of subjects would be enrolled and in a timely manner. This clinical trial was registered at ClinicalTrials.gov on May 14, 2018 (NCT03538561), just prior to enrollment of the first subject. The last subject's 30-day follow-up was May 15, 2019. NJF served as clinical monitor for the study.

Eligible patients were 21 years or older with an anesthetized 5-minute Schirmer score of ≤10 mm and a response to stimulation of 10 mm or more on the Schirmer strip. Complete inclusion and exclusion criteria are listed in Table 1. The inclusion and exclusion criteria were minimal so that a broad range of subjects would enter the study; however, the planning investigators felt that a Schirmer score of <10 mm would sufficiently identify subject who would both respond to stimulation and suffer from significant dry eye symptoms.

The study was conducted in compliance with current Good Clinical Practice guidelines and in accordance with the tenets of the Declaration of Helsinki. The study protocol was reviewed and approved as a non-significant risk study by the Quorum Review Institutional Review Board. Enrolled patients were required to sign a consent form prior to treatment.

**Table 1.** Inclusion and Exclusion Criteria

Inclusion Criteria	Exclusion Criteria
21 years of age or older	Sjögren's syndrome or other rheumatologic condition
Schirmer test with anesthetic of ≤10 mm/5 min in at least one eye	Intraocular surgery within 6 mo of visit 1
Ability to produce tears upon training with >10-mm change in Schirmer score compared to baseline in at least one eye	Intraocular or periocular injection within 6 mo of visit 1
In the opinion of the investigator, subject in good general health and free of any condition that could impair study participation or ocular evaluation	Used intranasal neurostimulation within 2 mo of visit 1 or planned to use it during the study
Subject willing and able to give written informed consent and commits to comply with study requirements	Lid function abnormalities
	Any acute infectious or non-infectious ocular condition of the anterior or posterior segments in either eye within 30 days of visit 1
	Diseases or conditions of ocular surface associated with clinically significant scarring or destruction of conjunctiva or cornea
	History of facial nerve palsy
	History of neuromuscular disorder
	Uncontrolled ocular or systemic disease
	Other clinically significant local skin condition (e.g., skin infection) at target treatment site
	Participation in any clinical trial with a new active substance or a new device within 30 days of visit 1 (with the exception of the devices to be used in the study described herein)

The primary assessment was change in 5-minute anesthetized Schirmer score assessed prior to (unstimulated) and after (stimulated) bilateral stimulation for 30 seconds on each side. Other assessments included the Ocular Surface Disease Index (OSDI), tear break-up time (TBUT), expressed meibomian gland analysis^9^ before and after stimulation, corneal and conjunctival staining, usability, and patient satisfaction.

Patients were encouraged to perform external nasal stimulation for 30 seconds on each side of the nose at least twice per day. Because this was a long-term follow-up study, subjects were followed closely with a phone call at day 3 and in-person office visits at days 14, 30, 90, and 180. An internal data logger on the device was used to monitor compliance.

### Statistical Methods

The statistical methods generally followed FDA guidance for past approval of the intranasal neurostimulator (ITN). The primary efficacy endpoint was the difference between the pre-stimulation and post-stimulation Schirmer score, also referred to as the Schirmer index. The worse eye was used in the analysis, also following convention. The statistical hypothesis for the primary endpoint was that the average Schirmer index at 30 days would be larger than zero when evaluated using a one-sided *t*-test with *P* ≤ 0.025. In addition, the two-sided 95% confidence interval (CI) for the mean was planned and based on the *t*-distribution. Data from an earlier pilot study at Olympic Ophthalmics and from the TrueTear device indicated that, with 24 subjects and given a SD of approximately 8 and actual difference of 9, there would be over 99% power to meet the primary efficacy endpoint. Additional related but unpowered endpoints included the proportion of subjects with Schirmer index scores of 5 mm and 10 mm. An intention-to-treat (ITT) analysis was performed for all subjects who enrolled in the study. For safety, all patients, including screen failures, were included. The hypothesis for the secondary endpoint is that the average change of OSDI from baseline is greater than zero at 30 days. Data from studies of the TrueTear ITN, which predicted a SD of 15 and a real difference of 12, were considered. Using these parameters, the study would have 99% power to show a difference from baseline for the primary and secondary endpoints with a sample size > 35. An adjustment for multiplicity was made in that the secondary endpoint would only be considered if the primary endpoint was statistically significant.

The number and percentage of subjects at each post-baseline assessment are presented, along with 95% exact binomial CIs. One hundred subjects were included in the study protocol so that a broad population of subjects would be studied, and we would be able to adequately determine safety and perform subgroup and exploratory analyses. Further analyses (unpowered) included comparison to the minimally clinically important difference (MCID) and proportion of subjects with various baseline severities and clinically significant differences in OSDI. Missing data for the primary endpoint were expected to be low and were studied as part of a sensitivity analysis. A single sensitivity analysis based on a multiple imputation approach to impute missing values as a function of the baseline Schirmer index value, the 14-day Schirmer index value, and baseline pre-stimulation score was used. Additionally, an analysis to assess the validity of pooling across sites and subject demographics was planned. The homogeneity of the 30-day mean Schirmer index score across the study sites was planned to be evaluated by site in a one-way analysis of variance. If the *F*-test *P* value associated with the site effect was less than or equal to 0.15, then the analysis would be considered indicative of site differences.

### iTEAR Device and Extranasal Nerve Stimulation

**Figure 1.**
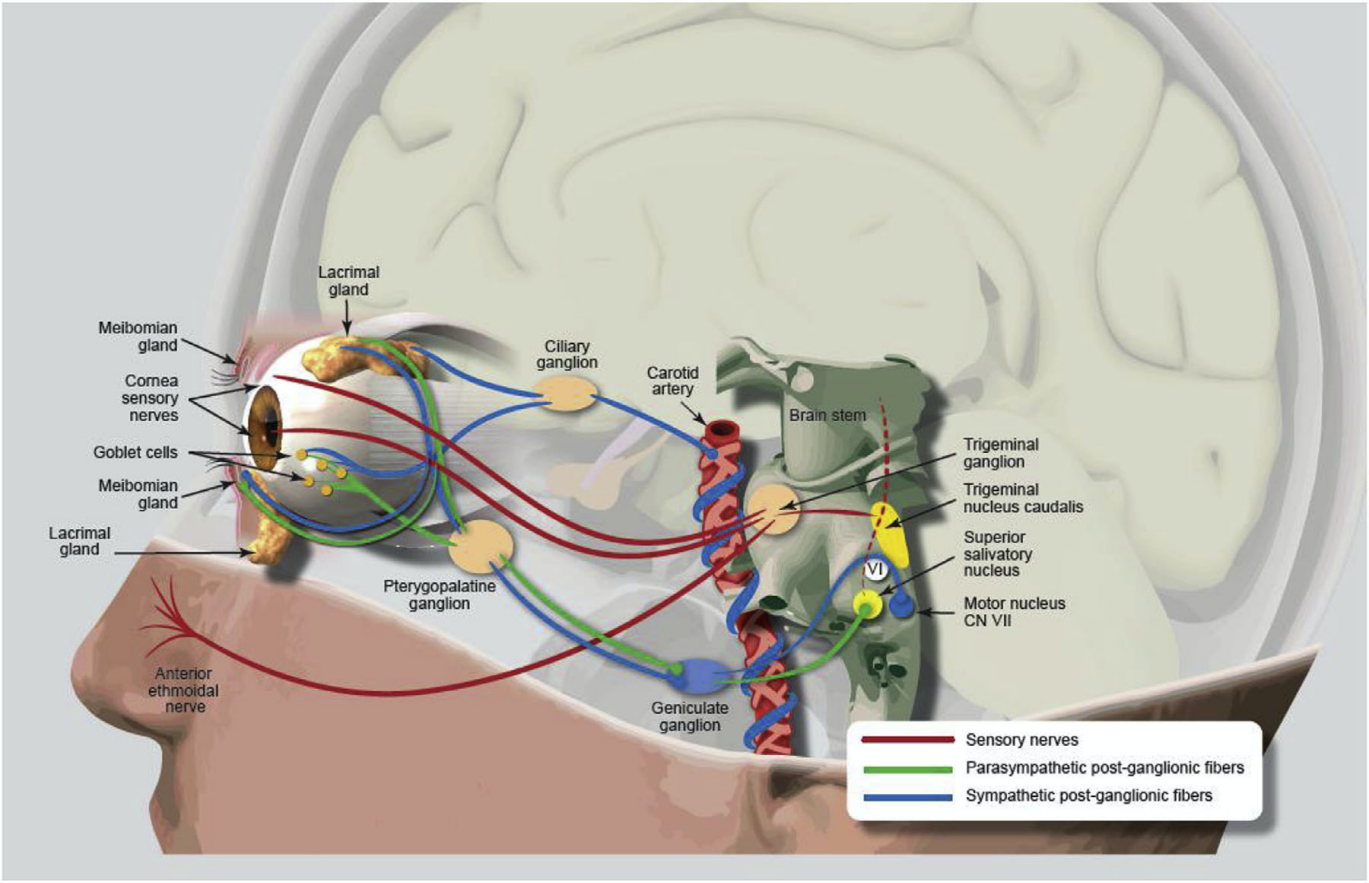
Pathway of the nasolacrimal reflex beginning with the anterior ethmoidal nerve or, as shown in this study, the external nasal nerve. Reprinted with permission from Dieckmann G, Fregni F, Hamrah P. Neurostimulation in dry eye disease-past, present, and future. *Ocul Surf*. 2019;17(1):20–27.

**Figure 2.**
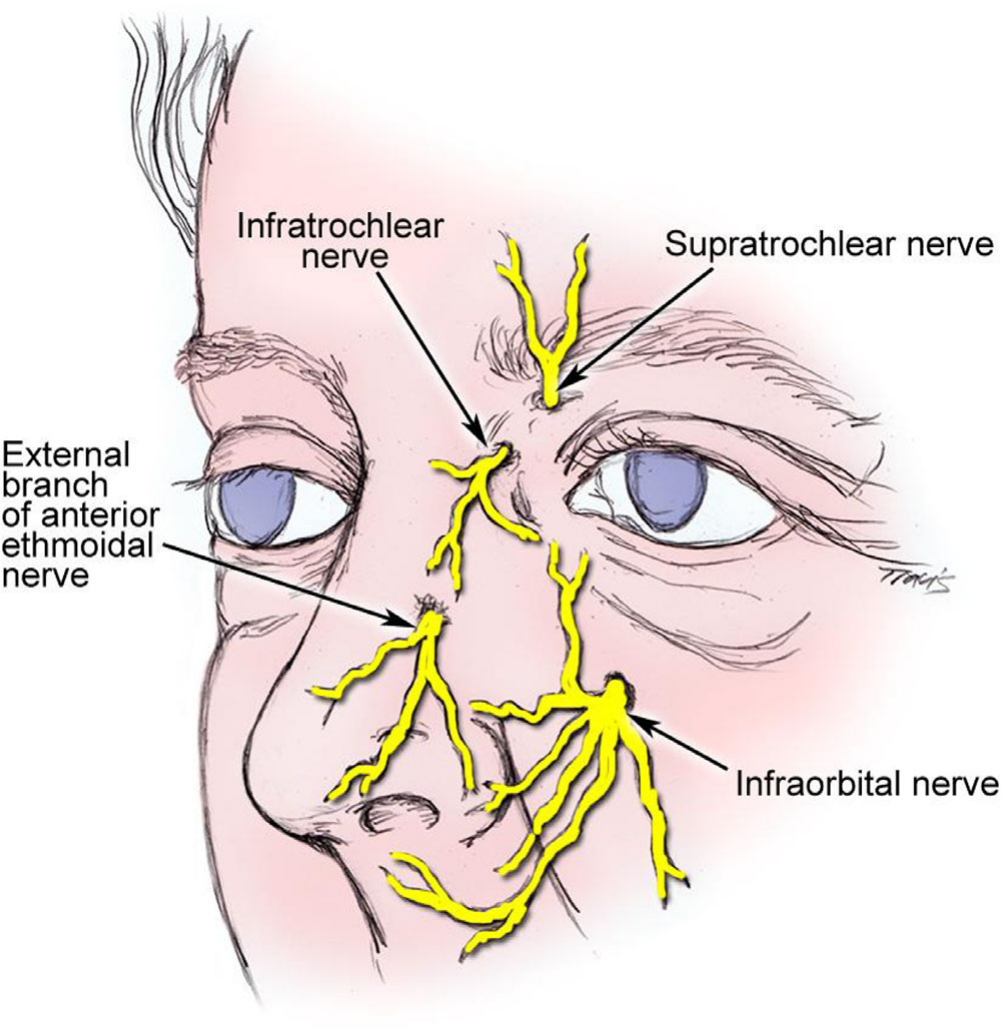
Location of the external branch of the anterior ethmoidal nerve (external nasal nerve). Reprinted with permission from Lal D, Gnagi SH. Nose anesthesia. *Medscape*, https://emedicine.medscape.com/article/82679-overview.

**Figure 3.**
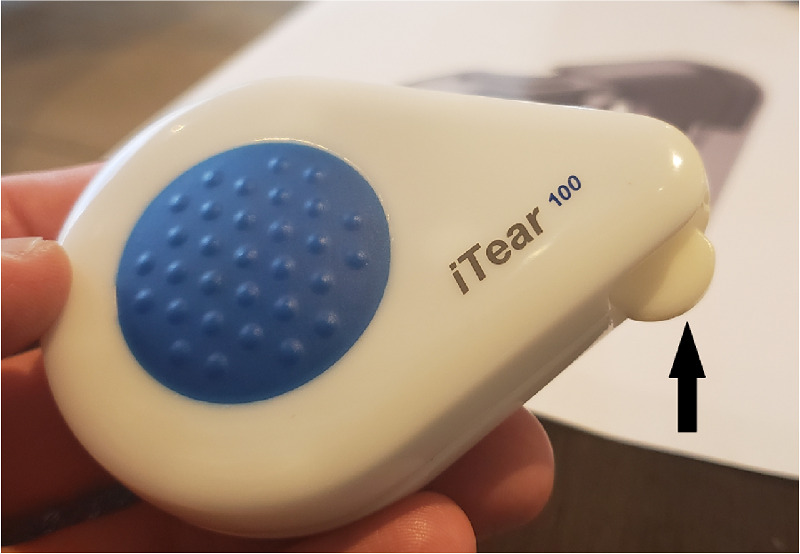
Commercial iTEAR100 device. The *arrow* denotes the oscillating tip with curvature and edge designed for chronic repetitive stimulation of the external nasal nerve.

Historically, the nasolacrimal reflex pathway begins with the anterior ethmoidal nerve, which connects to the trigeminal ganglion via the nasociliary and ophthalmic nerve (V1), ultimately communicating with the salivary nucleus and then the pterygopalatine ganglia (i.e., sphenopalatine ganglia) and, finally, the lacrimal nerve to stimulate the LFU (Fig. 1). The hypothesis underlying the iTEAR device is that the external nasal nerve (Fig. 2) also interfaces with this pathway through the nasociliary nerve. The external nasal nerve has previously been considered to be strictly a sensory nerve and has not been described as part of the nasolacrimal reflex, although it has been noted to communicate with the nasociliary nerve.^10^ To the knowledge of the authors, this is the first report showing that the external nasal nerve can be stimulated to activate the LFU by any means. Early work at Olympic Ophthalmics evaluated several parameters, including usability, optimal frequency, force, geometry, and durometer of the oscillating tip of iTEAR, to optimize treatment effect and minimize potential for skin damage, numbness, and other complications. The commercial version of the iTEAR device (iTEAR100) includes a unidirectional oscillating tip, with a frequency of approximately 220 to 270 Hz and an amplitude of roughly 0.5 to 1 mm (Fig. 3). It is placed against the skin of the nose around the junction between the nasal cartilage and the nasal bone where the external nasal nerve exits the skin onto the lateral aspect of the nose.

### Primary Outcome

As described in the statistical plan, the primary outcome was the Schirmer index at day 30. The study eye was defined as the eye with the lowest baseline pre-stimulation Schirmer score.

### Secondary Outcome

Severity of DED was classified according to subjective symptoms during the last week using the OSDI. Secondary outcome was the improvement of symptoms of DED evaluated using the OSDI at day 30. The OSDI was considered normal for values < 13, mild for values between 13 and 22, moderate for values between 23 and 32, and severe for values greater than 32. An OSDI increase of ≥22 was considered a marked change, and an increase of 13 was considered MCID in subjects with baseline OSDI ≥ 33.^11^

### Exploratory Outcomes

Additional exploratory endpoints, including the Schirmer index and OSDI, through day 180 were collected to further evaluate the study device and support its safety and effectiveness. Other exploratory outcomes included the Standard Patient Evaluation of Eye Dryness (SPEED) Questionnaire, eye dryness score (EDS), and meibomian gland expression and secretion quality using the Meibomian Gland Evaluator (Johnson & Johnson Vision, Santa Ana, CA), as well as the expression scale introduced by Korb,^9^ TBUT, and corneal and conjunctival staining evaluated using fluorescein dye along with the National Eye Institute (NEI) scale. Data for the fellow eye, the opposite one of the study eye, were also obtained.

### Location

Sites were chosen based on geographic diversity in order to obtain a broad variety of disease severity, demographics, eye care providers, and practice structures.

## Results

### Enrollment

Between May 2018 and April 2019, a total of 149 subjects were screened, 108 were enrolled, and 101 (93.5%) reached day 30. Analysis across sites did not indicate differences that would preclude pooling the data. Missing data for the primary endpoint were minimal, and sensitivity analysis for these missing data did not predict any meaningful difference. As far as screen failures, 41 out of 48 screen failures were due to a high baseline Schirmer score (>10 mm) while only 2/148 screened subjects (1.4%) were excluded from the study for inability to produce tears with neurostimulation. Seven subjects dropped out of the study prior to 30 days, which accounts for the missing data at the primary endpoint. One subject developed an unrelated methicillin-resistant *Staphylococcus aureus* infection, one reported nausea and dizziness after the first treatment, two did not think the device was helping, one experienced excessive ocular itching possibly due to the device, one did not follow up after the day 3 call, and one dropped out due to family issues. Eighty of the 101 subjects chose to continue beyond day 30, and 58 of them reached day 180. Table 2 summarizes the baseline characteristics at day 0. Fifty-nine subjects (54.6%) had a pre-stimulation Schirmer score of 5 mm or less, the average OSDI was 40, and over 80% of subjects used at least one treatment for DED.

Of the 108 subjects enrolled at baseline, 101 (94%) reached the primary endpoint at day 30. Eighty subjects chose to extend beyond 30 days, with 58 reaching day 180 (73%). There were no substantial differences between the groups as far as safety or effectiveness.

**Table 2.** Baseline Characteristics

Characteristic	*n*	Mean (SD)	Range
Schirmer score, pre-stimulation	108	6 (3.8)	0–23
Schirmer score, post-stimulation	108	28 (8.5)	7–35
Schirmer index (post-/pre-)	108	22 (7.8)	2–35
OSDI	105	40.3 (22.9)	2.1–92
Clear liquid secretion (pre-)	104	1.7 (3.1)	0–15
Clear liquid secretion (post-)	107	3.5 (4.9)	0–15
Meibomian gland (pre-), mean of two eyes	104	12.2 (10.2)	0–45
Meibomian gland (post-), mean of two eyes	107	17.5 (13)	0–45
Conjunctival staining of the worst eye	108	5.3 (4.4)	0–18
Corneal staining of the worst eye	108	3.4 (3.4)	0–15
TBUT of the worst eye	108	5 (3.6)	1.2–24
SPEED Questionnaire	108	14.1 (5.6)	1–27
EDS	103	56.8 (22.9)	0–100

### Outcomes

**Figure 4.**
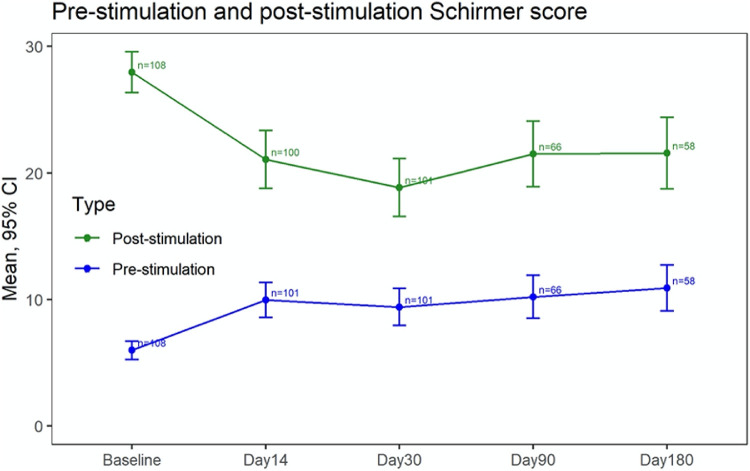
The primary endpoint of the study showing the pre-stimulation versus post-stimulation Schirmer score values.

There was a clinically and statistically significant change in Schirmer score following stimulation (Schirmer index) with iTEAR at day 0 and through day 30, the primary endpoint of the study (Fig. 4, Table 3). The mean Schirmer index ranged from 9.4 to 22.0 mm across the visits. At day 30 (the primary endpoint), the mean Schirmer index in 101 subjects was 9.4 mm, with 7.6 mm being the lower limit of the 95% CI. At day 30, the proportion of subjects with an increase in Schirmer score over 10 mm was 34%. In the 58 subjects who reached day 180, the mean Schirmer index was similar, with a value of 10.7 mm and lower CI limit of 8.3 mm.

From day 0 there was a clinically and statistically significant improvement of the pre-stimulation Schirmer score at every time point. In addition, the proportion of subjects with an increase of 5 mm or more on the pre-stimulation Schirmer compared to baseline values was over 30% at every time point (see Supplementary Table S3).

**Table 3.** Schirmer Scores and Index

	Schirmer Score, Mean (SD)				Schirmer Index			
Visit	Pre-Stimulation		Post-Stimulation		Mean (SD)	95% CI	*>*5, *n* (%)	>10, *n* (%)
Baseline	108	6 (3.8)	108	28 (8.5)	22 (7.8)	20.5–23.5	106 (98.1)	99 (91.7)
Day 14	101	10 (7.1)	100	21.1 (11.7)	11.1 (8.4)	9.4–12.8	66 (66)	40 (40)
Day 30	101	9.4 (7.6)	101	18.8 (11.8)	9.4 (9.3)	7.6–11.3	53 (52.5)	34 (33.7)
Day 90	66	10.2 (7.1)	66	21.5 (10.8)	11.3 (9.1)	9.1–13.5	40 (60.6)	30 (45.5)
Day 180	58	10.9 (7.1)	58	21.6 (11)	10.7 (9.1)	8.3–13	37 (63.8)	22 (37.9)

**Figure 5.**
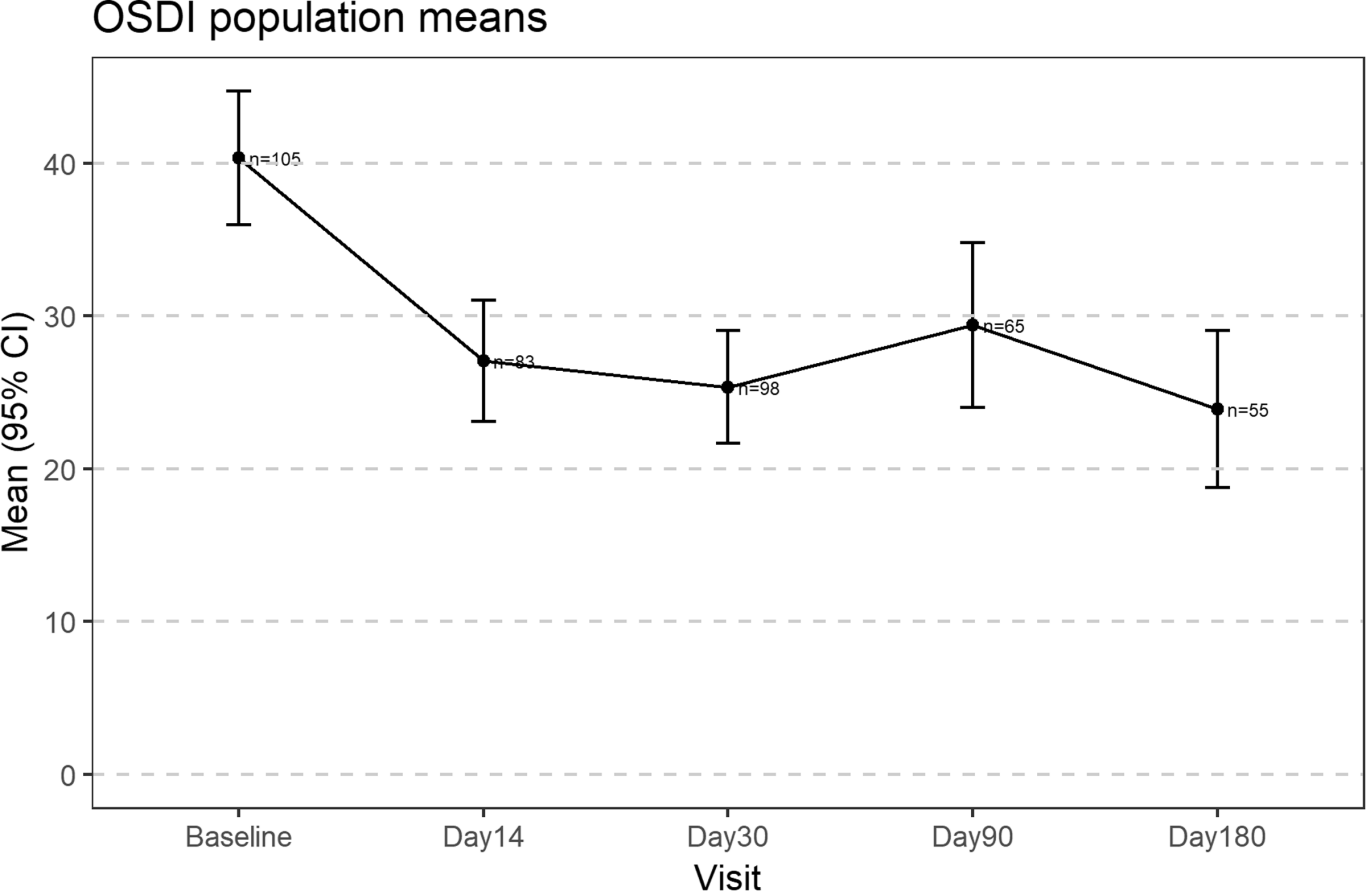
The secondary endpoint of the study showing OSDI scores at each time point.

Along with the Schirmer score changes, the OSDI with baseline as a comparison decreased significantly by an average of 14.4 at day 30 (95% CI, 11.1–17.7). The change was statistically and clinically significant from the first follow-up visit at day 14 through day 30 (Fig. 5, Table 4). Additionally, 64 patients (66.7%) reached MCID and 29 had marked improvement (30.2%) at day 30, whereas among subjects with severe DED 30.2% experienced MCID (Table 5). These percentages of responses remained constant out to Day 180.

Pre-stimulation and post-stimulation Schirmer scores for the fellow eye, as well as its Schirmer index, were almost identical to the values of the study eye throughout the entire study period (see Supplementary Table S15). The remaining list of exploratory endpoints can be found in the Supplementary Materials.

**Table 4.** Ocular Surface Disease Index

	OSDI		OSDI Change from Baseline	
Visit	*n*	Mean (SD)	Mean (SD)	95% CI
Baseline	105	40.3 (22.9)	–	–
Day 14	83	27.1 (18.5)	–14.2 (14.9)	(–17.5, –10.9)
Day 30	98	25.4 (18.6)	–14.4 (16.1)	(–17.7, –11.1)
Day 90	65	29.4 (22.2)	–13.7 (18.7)	(–18.4, –9.1)
Day 180	55	23.9 (19.4)	–19.3 (18.6)	(–24.3, –14.3)

**Table 5.** Response Groups Based on OSDI Change

	*n* (%)		
Day	Overall MCID^a^	MCID in Severe DES^b^	Marked Improvement^c^
14	51 (62.2)	44 (57.1)	26 (31.7)
30	64 (66.7)	48 (64.0)	29 (30.2)
90	41 (63.1)	32 (59.3)	21 (32.3)
180	37 (67.3)	32 (68.1)	23 (41.8)

^a^Improvement of 8 OSDI points or more.

b Improvement of 13 OSDI points or more.

c Improvement of 22 OSDI points or more.

### Safety and Compliance

No serious adverse events related to the device occurred throughout the study. Two adverse events of mild severity were definitely related to the device, and seven adverse events possibly related to the study device were recorded (see Supplementary Tables S26 and S27). Importantly, there were no neurologic adverse events related to the cranial nerves or damage to the skin. There was one incidence of “nasal pain,” but this pain did not affect the subject's use of the device throughout the trial. The device logger showed over 27,000 device applications in the trial, for an average of close to the recommended two applications per day during the first 30 days and every other day for those subjects who completed 180 days. Concomitant medication usage, as determined from daily diaries, also indicated compliance, although many subjects decreased usage of artificial tears. Using information from daily diaries, 44% of the subjects who were using artificial tears at the study outset decreased their usage and 23% stopped altogether.

### Patient Satisfaction and Usability

Patient satisfaction and usability (subject assessment and investigator observation) surveys were performed to further assess the performance of the device after its first use. Each subject was asked to read the instructions for use and were trained briefly (∼30 seconds) before the first use. Subjects were naïve to the device at the time of the surveys and were first trained on the device at the initial visit. Close to 100% of users found the device to be easy to use (see Supplementary Table S19). Two percent of subjects were judged by the investigator to not be able to find the external nasal nerve or hold the device in the correct position for 30 seconds (see Supplementary Table S20). Subject satisfaction at 30 days showed that 41% of subjects were “very satisfied,” 40% “satisfied,” 16% “neither satisfied nor dissatisfied,” and 4% “dissatisfied” (see Supplementary Table S21). The four patients who reported being dissatisfied were compliant with the treatment but did not notice improvement in their symptoms, which was the reason for their dissatisfaction. As far as signs in these subjects, one subject had improved corneal staining, and another one had an improvement in basal Schirmer score. The other two subjects appeared to have no improvement in signs or symptoms.

## Discussion

The hypothesis in this clinical trial was that using mechanical oscillation to stimulate the external nasal nerve, which emanates from the region around the junction between the nasal cartilage and the nasal bone, leads to activation of the nasolacrimal reflex, a pathway typically referred to in the context of intranasal activation. This study demonstrates that stimulation of this novel target with the iTEAR100 device from Olympic Ophthalmics results in immediate tear production, improvement in basal tear production, improvement in symptoms, and an acceptable safety profile. These data for iTEAR are consistent through the primary endpoint of 30 days and through the 180-day extended exploratory endpoints, as well. Additional outcomes, including patient satisfaction and usability, are supportive of the commercial potential of the iTEAR device. The exploratory endpoints, including meibomian gland expression assays, TBUT, and corneal and conjunctival staining, all support the hypothesis that extranasal stimulation of the nasolacrimal reflex via the external nasal nerve results in improvement of the entire LFU and, in general, the signs and symptoms of DED.

Neuromodulation is a decades-old device concept but has only recently been proposed for ophthalmic disorders. Its theoretic underpinnings are based on modifying neuronal function through stimulation using drugs or electrical or magnetic or mechanical signals. Neurostimulation devices have been approved by the FDA to treat a wide variety of diseases, mostly in the neuropsychiatric field, including Parkinson's disease, chronic neuropathic pain, overreactive bladder, major depressive disorder, obsessive–compulsive disorders, movement disorders, and epilepsy.^12^^–^^18^ The clinical effects of many of these devices are not limited to the time of stimulation only. There is a build-up of effect with chronic use. Indeed, the data presented in this study suggest improved basal secretion from the LFU.

The nasolacrimal reflex plays an important role in aqueous tear production and is triggered by nasal airflow, which accounts for 34% of the basal lacrimal secretion.^6^ Lacrimal glands are predominantly innervated by the parasympathetic branch of the autonomous nervous system, whereas the impact of the sympathetic branch on lacrimal secretion is still poorly understood. Specifically for DED, the first portable ITN, TrueTear, was approved by the FDA in 2017 for the treatment of DED in adults. TrueTear, through two intranasal electroconductive tips, provides activation of the nasolacrimal reflex, resulting in an increase of tear production. With four-times daily application of the device, signs of DED including Schirmer score, conjunctival (but not corneal) staining, and symptoms measured using OSDI score and the dry eye symptom visual analog scale, were markedly improved at day 180 compared to baseline.^8^ ITN provides symptomatic relief for an average of 3 hours. Further studies of the TrueTear device confirmed its efficacy for the treatment of DED using additional parameters, including tear meniscus height, degranulation of conjunctival goblet cells, and their area.^19^^–^^27^ The effects of the ITN device on corneal staining as well as the protein and lipid component of tears differ across various studies,^8^^,^^20^^,^^22^^,^^24^^–^^27^ warranting further investigation. Despite its efficacy and safety for the treatment of DED, the ITN device requires deep insertion into the nose, which makes it not practical or comfortable for many patients.

In this study, basal tear production increased along with acute tear production after stimulation (Schirmer index). The decrease in the magnitude of the Schirmer index over time is likely due to development of partial tolerance to neurostimulation and simultaneous increase in basal tear production. Tolerance can develop quickly, even as early as the day 3 phone call. Similarly, after the first follow-up visit, a tolerance is seen with ITN, as well.^8^ One has to be careful about interpreting the tolerance and the Schirmer score and index, as they are surrogate markers for tearing and corneal surface changes. An acute change of >10 mm on Schirmer paper indicates obvious wetting that is not necessarily representative of what might be required for symptom improvement in a real-world setting. That is, clinical benefit may be derived from small changes in the tear film for which there is minimal change of Schirmer index; therefore, Schirmer-based tolerance should be distinguished from clinical tolerance. Indeed, in this study the symptom scores and other signs indicated continued benefit in subjects with tolerance based on Schirmer score.

The OSDI is a patient-reported outcome (PRO) score designed to provide a rapid assessment of the range of ocular surface symptoms related to chronic DED.^11^ This PRO score has been used in numerous trials and approvals for over 20 years. The concept of MCID is typically referred to for interpretation of changes in a PRO to provide clinical relevance of the change in score. Based on their trial and literature review, Miller et al.^11^ suggested that a change from baseline of approximately 8 is clinically significant when subjects with any baseline OSDI are considered, and a change of 13 is clinically significant when only severe subjects are considered (OSDI ≥ 33). These parameters were used to determine the clinically important change in OSDI in this study. The results show that, in subjects with severe DED, 75% showed significant improvement based on the OSDI measurement at day 30.

There has been increasing interest in the development of devices and drugs that can improve meibomian gland function. The secretion from these glands, called meibum, constitutes the lipid layer of the tear film, and it prevents evaporation of the aqueous component. Meibomian gland dysfunction (MGD) is often present as a comorbidity in the majority of patients with DED^28^ and has an intricate relationship with it, especially the evaporative subtype. DED can alter meibomian gland structure and function, but at the same time MGD contributes to increasing the severity of DED. Previous studies with the TrueTear ITN yielded mixed results regarding effect on meibomian glands, although the assessment techniques were heterogeneous.^22^^,^^24^^,^^25^ In our study, meibomian gland expression was an exploratory endpoint, as the study investigators noticed increased expression from the glands with neurostimulation in early proof of concept. We used an assessment technique similar to that used in studies of TearScience LipiFlow (Johnson & Johnson Vision), whereas the methods for TrueTear studies have relied on quantitative meibography. The data in our study show an increase in pre-stimulation expression and meibum quality by day 30 (see Supplementary Tables S8 and S9). There was minimal change in expression immediately after stimulation despite investigators’ qualitatively documenting immediately increased secretion. The approach to measuring gland expression immediately after stimulation has not been fully established. In this study, the post-stimulation expression was performed within 15 minutes of the first expression and within 5 minutes of the stimulation. It is possible that the immediate re-expression does not allow sufficient time for additional oils to re-accumulate in the meibomian glands. It is also possible that the expression test used for the evaluation of LipiFlow is not appropriate for iTEAR. The expression output parameters were greater when the data were subgrouped into baseline >12 or ≤12 to match the inclusion criteria of the LipiFlow studies.^29^ The data notwithstanding, the effect of neurostimulation on meibum secretion is exploratory and not conclusive, requiring further study.

TBUT improved significantly, as well. The results after day 30 were not statistically significant, but the statistical power in this second phase of the study was lower due to the smaller number of subjects in the second phase.

Corneal and conjunctival staining typically have parallel improvements in the ocular surface. In this study, staining was performed at each follow-up visit prior to stimulation. An improvement was seen by day 14, the improvement being more dramatic in subjects with higher baseline scores, which represent more severe disease. As with the other exploratory endpoints, corneal and conjunctival staining data are suggestive but would have to be studied as primary endpoints in their own right to draw definitive conclusions.

The iTEAR device is safe and able to induce acute lacrimation and significantly ameliorate signs and symptoms of DED. Further data are needed to assess long-term effectiveness and safety. One limitation of this study is the absence of a control or sham group; however, the primary endpoint of immediate tear production onto a Schirmer strip is not likely to be affected by placebo or Hawthorne effect. Indeed, a separate single-day study (unpublished) showed that a sham device that made noise but did not impart energy to the skin did not result in any tear production above the basal tearing on the Schirmer strip. The OSDI and other symptom scores are susceptible to Hawthorne and placebo types of effects. The OSDI, in particular, has been validated over many years and a decrease of >8 and certainly >13 is widely accepted as evidence of clinical benefit. The meibomian gland expression score and the increase in basal tears were not expected benefits at the outset of the study based on available data for the ITN. These endpoints are newer to studies of DED; therefore, future studies will utilize a control to further understand the clinical effect size.

## Supplementary Material

Supplement 1
