# Supplementary material for: Novel Extranasal Tear Stimulation: Pivotal Study Results
Source: Transl Vis Sci Technol. 2020 Nov 17;9(12):23. doi: 10.1167/tvst.9.12.23 (PMC7683850; doi:10.1167/tvst.9.12.23)
Supplement: Supplement 1 [file tvst-9-12-23_s001.pdf]

Supplementary Appendix

This appendix has been provided by the authors to give readers additional information about their work.

Supplement to: Novel Extranasal Tear Stimulation: Pivotal Study Results

Contents

List of principal investigators and participating sites 2

Additional Baseline Data 3

Exploratory Endpoints 4

Pre-stimulation Schirmer score 4

Ocular Surface Disease Index (OSDI) values and change by baseline OSDI severity 5

Standard Patient Evaluation of Eye Dryness (SPEED) and Eye dryness Scale (EDS) 6

Meibomian Gland Expression 6

Gland Secretion Quality 7

Meibomian Gland subgroups analysis 8

Tear Break Up Time (TBUT) 9

Corneal and Conjunctival staining 9

Schirmer Score of the fellow eye 10

Habituation 11

Pain and Discomfort 12

Global Assessment of Change 12

Usability 13

Satisfaction 13

Recommend to friend 14

Use Again 14

Compliance 15

Adverse Events 16

List of principal investigators and participating sites

Investigator	Clinic	Enrolled participants
Ann Jayaram MD	Mid Peninsula Ophthalmology Medical Group (Menlo Park, CA)	12
Laura Periman MD	Evergreen Eye Center (Seattle, WA)	9
Paul Karpecki OD	Kentucky Eye Institute (Lexington, KY)	7
David Kading OD	Eye Specialty Eyecare Group (Seattle, WA)	24
Cynthia Matossian MD	Matossian Eye Associates (Pennington, NJ)	20
Andy Mu OD	NV Eye Surgery (Las Vegas, NV)	14
Gerald Walman MD	Walman Eye Center (Sun City, AZ)	17
Scott Markham DO	M&M Clinic (Prescott, AZ)	5

Additional Baseline Data

Table S1: Demographic characteristics of eligible subjects

		n
Sex	F	82
	M	26
Age	<30	6
	30-50	18
	50-70	60
	>70	24
Ethnicity	Hispanic/Latino	5
	Not Hispanic/Latino	101
	N/A	2
Race	White	87
	Black	2
	Other	3
	N/A	16
	Total	108

Table S2: Medication use at baseline

Medication	n
Artificial tears (AT)	54
AT + anti-inflammatory (Xiidra/Restasis)	24
Anti-inflammatory only	4
None	19

Exploratory Endpoints

Pre-stimulation Schirmer score

Schirmer test was used to evaluate the primary and exploratory endpoints. Topical anesthetic drops were instilled in both eyes of the subject. After 2 min (1 min with eyes closed and 1 min with eyes open), color bar Schirmer strips (Katena Parsippany, NJ) were placed in each eye at the junction of the middle and lateral thirds of the lower eyelid. The subject was instructed to keep his/her eyes closed and not talk. The test was performed in a dimly lit room with no direct air on the subject's face. After 5 min, the amount of basal wetting was recorded, and the device applied for up to 30 sec per side with the same strip in place. After an additional 4 min, the additional wetting was recorded. The difference in Schirmer wetting from baseline to post stimulation served as the measure of Schirmer Index, the primary endpoint. In many cases, the strip fully wet before the 5 min and the maximum 35 mm recorded as the post-stimulation value. Because all subjects had a low Schirmer score to begin with, there was very little movement due to reflex tear after the basal test. Unpublished pilot data showed that maintaining the same strip in place was preferable to variabilities of changing to a second strip and inability to standardize the time of strip insertion relative to device application.

Table S3: Change in pre-stimulated Schirmer score

Visit	N	Mean (SD)	CI 95%	>5 (%)
Day14	101	3.9 (6.3)	2.6, 5.1	31 (30.70)
Day30	101	3.3 (7.4)	1.9, 4.8	32 (31.70)
Day90	66	4.4 (6.5)	2.8, 6.0	23 (34.80)
Day180	58	4.9 (5.6)	3.4, 6.3	25 (43.10)

Ocular Surface Disease Index (OSDI) values and change by baseline OSDI severity

The Ocular Surface Disease Index (OSDI) is a 12-item questionnaire used to assess symptoms of dry eye. OSDI was considered normal if <13, mild if between 13 and 22, moderate if between 23 and 32, and severe if >32. OSDI improved more in the moderate and severe subgroups.

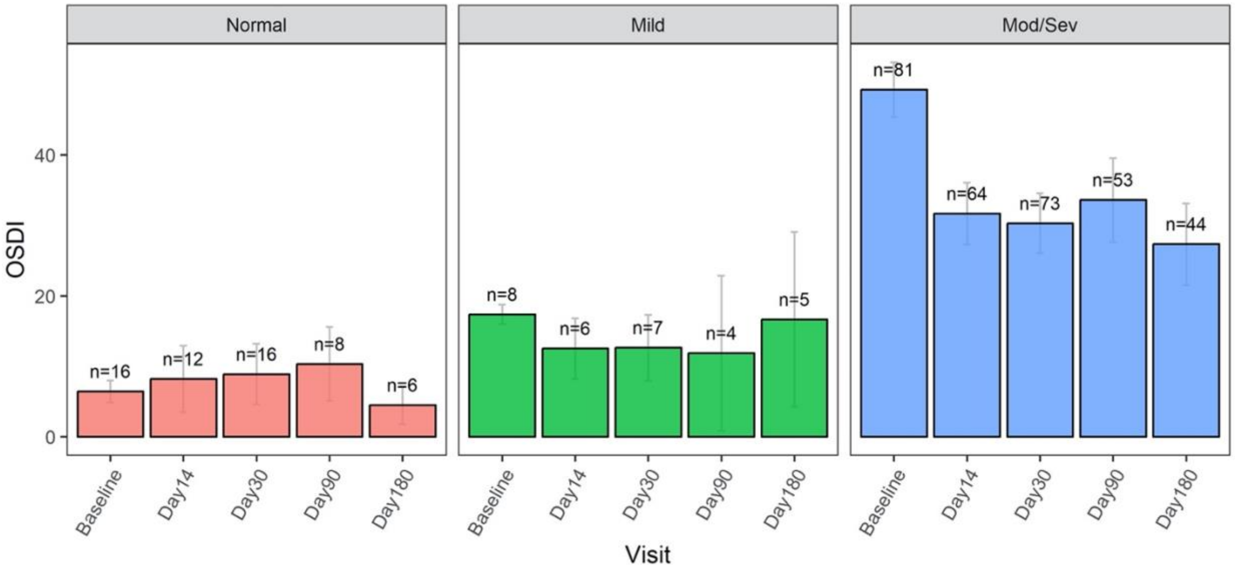

Figure S4: OSDI values by baseline OSDI severity

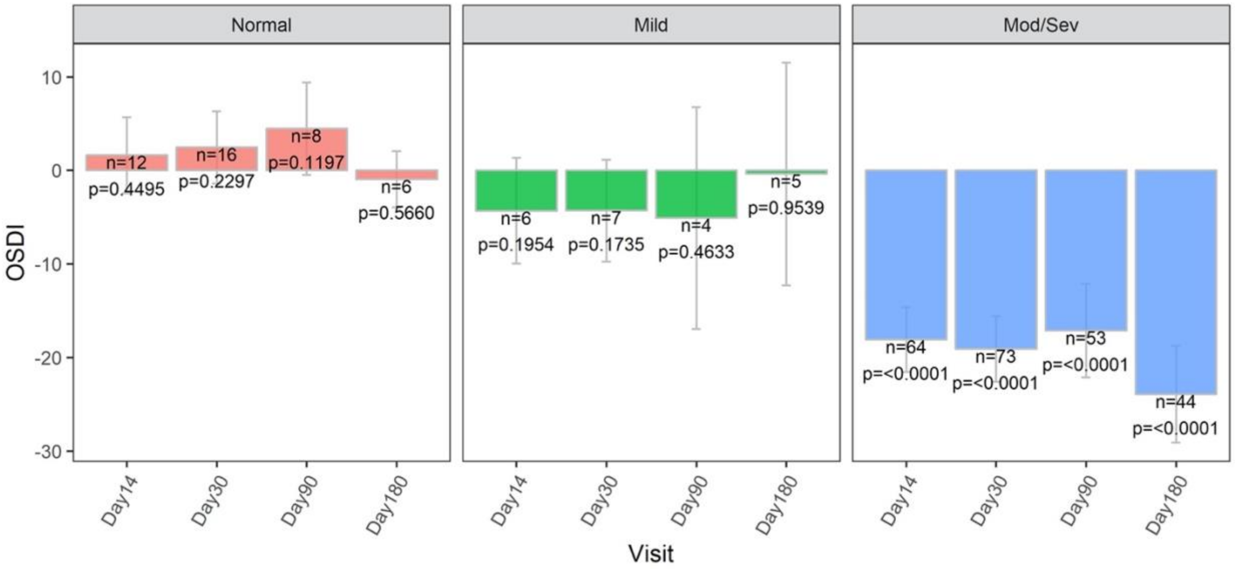

Figure S5: Change in OSDI by baseline OSDI severity

Standard Patient Evaluation of Eye Dryness (SPEED) and Eye Dryness Scale (EDS)

Standard Patient Evaluation of Eye Dryness (SPEED) and Eye Dryness Scale (EDS) are two methods used to assess the severity of DES symptoms. SPEED is a questionnaire and a score greater than 9 is generally considered moderate to severe dry eye whereas a 2-3 point change is a clinically significant difference. EDS is a visual analogue scale where 0 corresponds to no discomfort and 100 to maximal discomfort. Population means along with change from baseline were measured. Mean Change represents the mean difference between the time point and day 0, the average change for all subjects at that time point.

Table S6: SPEED

Visit	n	Mean (SD)	Mean Change (SD)	CI 95%
Baseline	108	14.1 (5.6)	N/A	N/A
Day 14	102	10.7 (5.3)	-3.3 (4.2)	−4.1, −2.5
Day 30	100	10.1 (5.3)	-4 (4.9)	−5.0, −3.0
Day 90	66	9.8 (5.6)	-5 (5.1)	−6.2, −3.7
Day 180	56	9.3 (5.4)	-5.5 (6)	−7.1, −3.9

Table S7: EDS

Visit	n	Mean (SD)	Mean Change (SD)	CI 95%
Baseline	103	56.8 (22.9)	N/A	N/A
Day 14	102	38.7 (21.9)	-17 (24.7)	−22.0, −12.1
Day 30	101	35.9 (21.5)	-19.3 (24.7)	−24.3, −14.3
Day 90	66	34 (23.1)	-23.2 (27.4)	−30.1, −16.2
Day 180	56	34 (23)	-22.8 (23.7)	−29.3, −16.3

Meibomian Gland Expression

The meibomian gland expressor (Johnson & Johnson Surgical Vision Incorporated, Santa Ana, CA) was used along with the expression scale devised by Korb. Expression was graded on a scale of 0 to 45 and each of 15 glands in three regions (temporal, central and nasal) was graded on a scale of 0 to 3. Clear liquid secretion is the highest level of secretion, given the number 3. Population means as average of both eyes along with change from baseline were measured. Mean Change represents the mean difference between the time point and day 0, the average change for all subjects at that time point.

Table S8: Meibomian Gland Expression before and after stimulation

	Expression, pre				Expression, post				
Visit	n	Mean (SD)	Mean Change (SD)	p value	n	Mean (SD)	Mean Change (SD)	p value
Baseline	104	12.2 (10.2)	N/A	N/A	107	17.5 (13)	N/A	N/A
Day 14	102	16.4 (12.2)	4.2 (10.4)	<0.0001	100	19.2 (13.5)	1.2 (10.3)	0.2444
Day 30	101	19 (13.5)	6 (11.6)	<0.0001	101	21.3 (15)	3.5 (12.1)	0.0043
Day 90	66	20.6 (13.7)	7.8 (14.6)	<0.0001	65	23.1 (15)	4.3 (13.8)	0.0145
Day 180	58	18.9 (14.9)	4.6 (14.2)	0.0186	58	20.7 (15.1)	0.8 (13.2)	0.6605

Gland Secretion Quality

Evaluation of clear liquid secretion for the 15 analyzed Meibomian glands. An increase of 1.5 at 30 days means that 1.5 out of 15 glands in one eye produces clear liquid as compared to the baseline. As in other studies, 1-2 additional glands is clinically significant. Population means of as average of both eyes along with change from baseline were measured. Mean Change represents the mean difference between the time point and day 0, the average change for all subjects at that time point.

Table S9: Clear liquid secretion

	Pre-stimulation clear liquid secretion				Post-stimulation clear liquid secretion			
**Visit**	**n**	**Mean (SD)**	**Mean Change (SD)**	**p value**	**n**	**Mean (SD)**	**Mean Change (SD)**	**p value**
Baseline	104	1.7 (3.1)	N/A	N/A	107	3.5 (4.9)	N/A	N/A
Day 14	102	3.2 (4.3)	1.5 (3.7)	<0.0001	100	4.1 (5)	0.5 (3.2)	0.1620
Day 30	101	4.1 (5.1)	2.1 (4.5)	<0.0001	101	4.7 (5.8)	1.2 (4.1)	0.0052
Day 90	66	4.6 (5.1)	2.7 (5)	<0.0001	65	5.5 (5.8)	1.6 (4.9)	0.0090
Day 180	58	4.2 (5.1)	1.9 (4.3)	0.0017	58	4.8 (5.6)	0.7 (3.8)	0.1801

Meibomian Gland subgroups analysis

To evaluate the efficacy of the device in the aqueous deficiency subtype compared to the evaporative subtype, the study population was divided into two subgroups based on meibomian expression score. Patients with score ≤12 were considered aqueous deficiency subtype and >12 were considered evaporative subtype. The number 12 was selected because it served as the inclusion criteria in studies which evaluated the Lipiflow device.

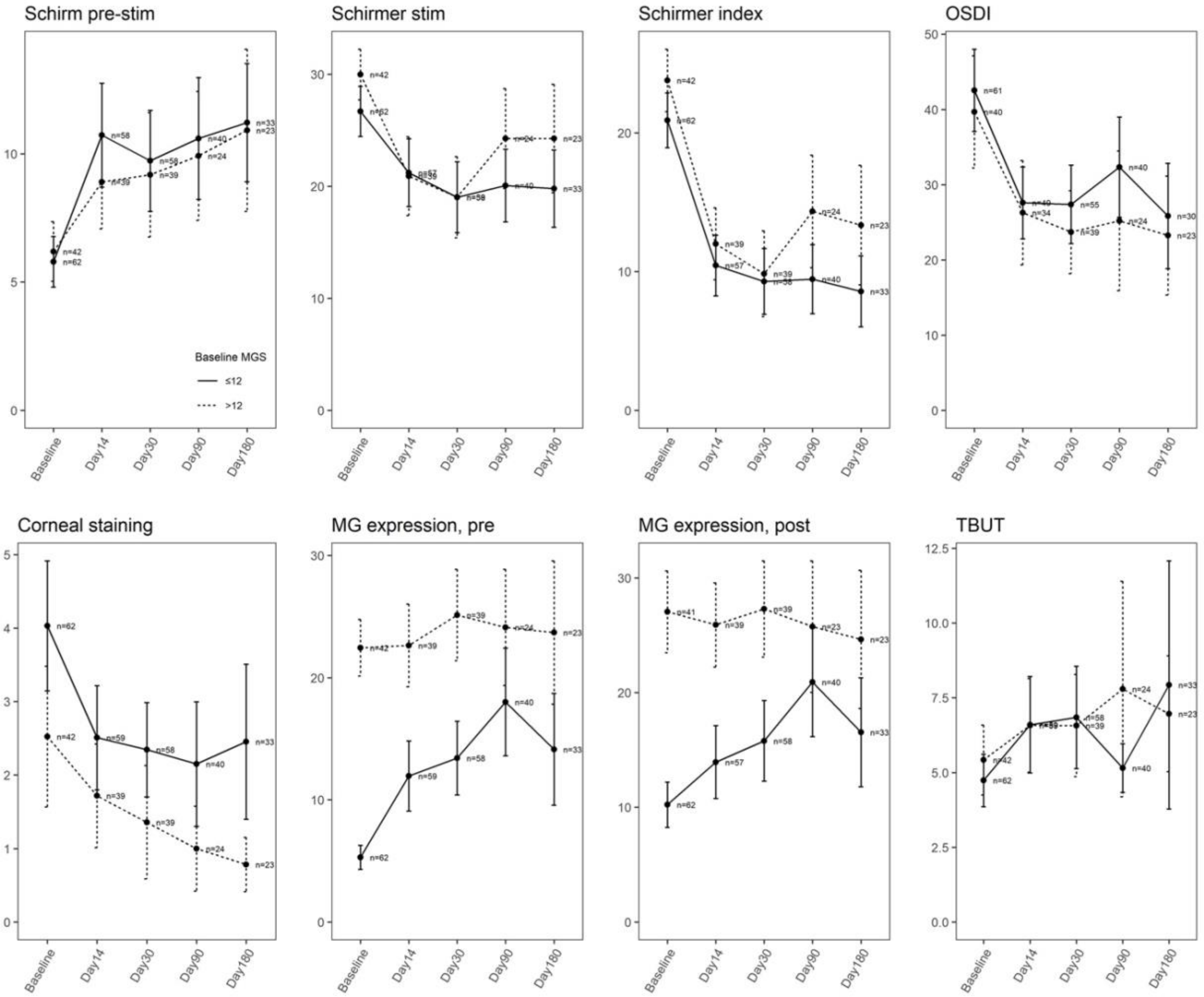

Figure S10: Meibomian gland subgroup analysis of various lacrimal parameters

Tear Break Up Time (TBUT)

Tear Break Up Time (TBUT) is a measure of the lipid layer and overall tear film quality. After fluorescein instillation the subject is asked to hold their eyes open, and the time for the tear film to break up is quantified. TBUT was measured as the time between the last blink and the appearance of one or more black (dry) spots in the pre-corneal tear film. The measurement was repeated twice, and average was taken. If the two measurements differed by more than 10%, a third measurement was taken. A lower time indicates a poorer quality tear film with 6s generally considered the normal cut off. Clinically significant change in other studies has been 1-2 sec on average. Mean Change represents the mean difference between the time point and day 0, the average change for all subjects at that time point.

Table S11: TBUT

Visit	n	Mean (SD)	Mean Change (SD)	p value
Baseline	108	5 (3.6)	N/A	N/A
Day 14	99	6.6 (5.8)	1.5 (4.5)	0.0013
Day 30	101	6.7 (6)	1.7 (5.6)	0.0035
Day 90	66	6.2 (5.9)	1 (5.8)	0.1653
Day 180	58	7.5 (9.6)	2.2 (9.2)	0.0802

Corneal and Conjunctival staining

Corneal and conjunctival staining using National Eye Institute (NEI) grading nomenclature was used. This scoring system divides the corneal and conjunctival surface into respectively five and six sections and assigns a value from 0 to 3 to each section. Population means of the worst eye along with change from baseline, and the change from baseline in the subgroup with >3 baseline staining were measured. Mean Change represents the mean difference between the time point and day 0, the average change for all subjects at that time point.

Table S12: Corneal staining

Visit	n	Mean (SD)	Mean Change (SD)	p value
Baseline	108	3.4 (3.4)	N/A	N/A
Day 14	102	2.1 (2.6)	-1.2 (2.4)	<0.0001
Day 30	101	1.9 (2.5)	-1.4 (2.9)	<0.0001
Day 90	66	1.7 (2.4)	-1.8 (3.1)	<0.0001
Day 180	58	1.8 (2.5)	-1.6 (3.1)	0.0003

Table S13: Conjunctival staining

Visit	n	Mean (SD)	Mean Change (SD)	p value
Baseline	108	5.3 (4.4)	N/A	N/A
Day 14	102	3.5 (3.4)	-1.9 (3.8)	<0.0001
Day 30	101	3.8 (3.6)	-1.3 (4.2)	0.0020
Day 90	66	3.3 (3.8)	-1.5 (3.7)	0.0013
Day 180	58	2.5 (2.7)	-2.1 (3.8)	0.0001

Table S14: Change in corneal and staining in the subgroup with baseline >3

	Change in corneal staining			Change in conjunctival staining		
**Visit**	**n**	**Mean Change (SD)**	**p value**	**n**	**Mean Change (SD)**	**p value**
Day 14	34	-3.1 (2.8)	<0.0001	59	-3.2 (4.2)	<0.0001
Day 30	33	-4 (2.6)	<0.0001	57	-3 (4.4)	<0.0001
Day 90	24	-4.5 (2.7)	<0.0001	33	-3.1 (4.5)	0.0004
Day 180	21	-4.1 (3.2)	<0.0001	30	-3.8 (4.5)	<0.0001

Schirmer Score of the fellow eye

Almost identical data to the study eye indicates that both eyes in the subject are being treated and the response carries over to the fellow eye.

Table S15: Schirmer value in fellow eye

	Pre-stimulation value		Post-stimulation value		Schirmer Index
**Visit**	**n**	**Mean (SD)**	**n**	**Mean (SD)**	**Mean (SD)**
Baseline	108	7.8 (5.4)	108	28.4 (9.8)	18.5 (9)
Day 14	101	10.9 (7.5)	100	20.9 (11)	9.3 (7.4)
Day 30	101	10.1 (7.7)	101	19.6 (10.9)	8.6 (8.7)
Day 90	66	10.9 (8)	66	22.2 (11.4)	10.6 (8.9)
Day 180	58	12.1 (7.9)	58	23.9 (11.3)	10.8 (9.5)

Habituation

"Habituator" is defined two ways. At "Day 30," habituator group includes subjects whose Schirmer index was <10 and therefore would not have qualified for the study initially. "Day 14" is a more stringent definition of habituator in which the index was <5 by day 14.

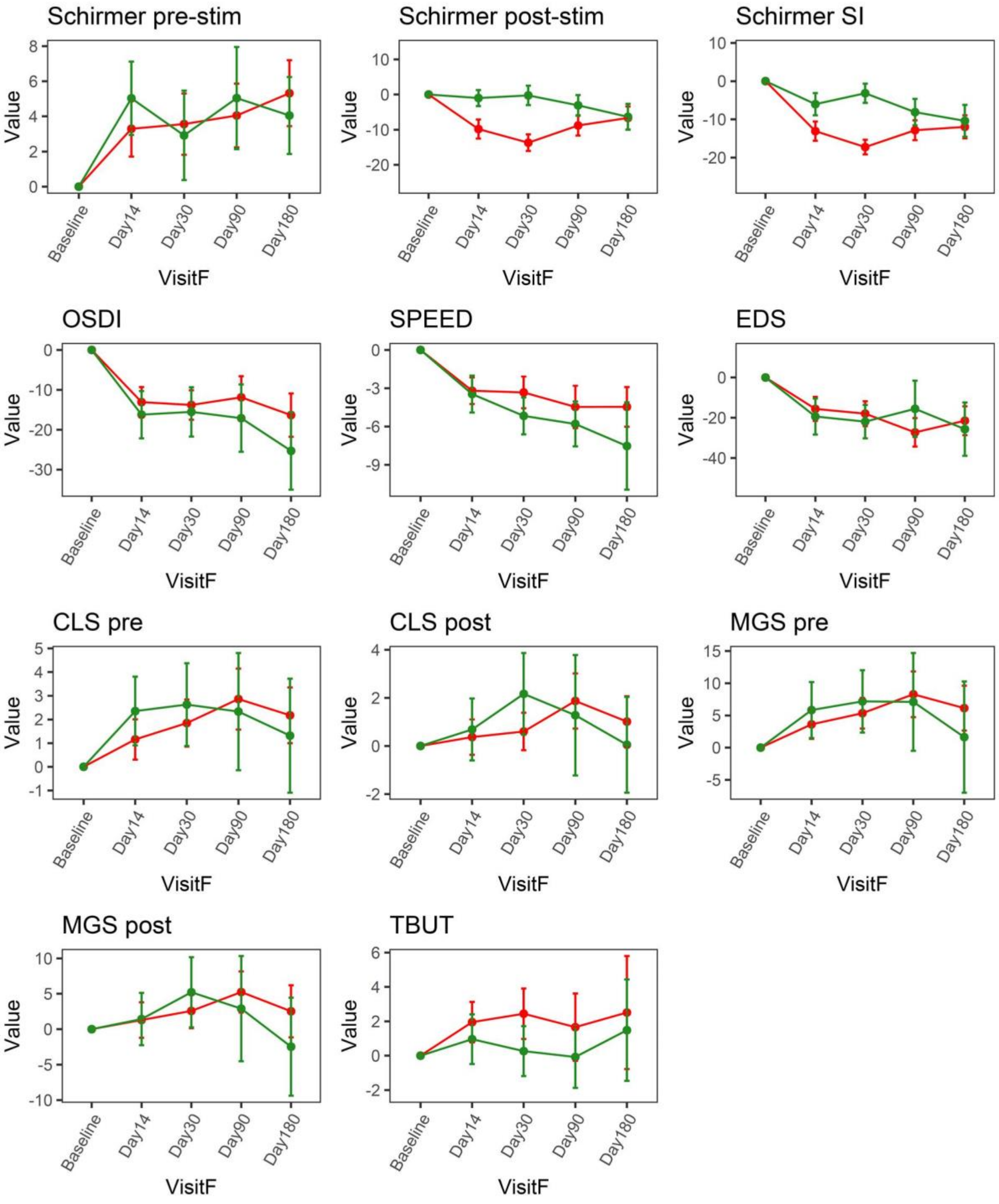

Figure S16: Change scores comparing habituators (red) and non-habituators (green)

Pain and Discomfort

Pain and discomfort were assessed with a modified Wong-Beker FACES® scale

Table S17: Pain and discomfort rating

	No hurt	Hurts little bit	Hurts little more	Hurts even more	Hurts worst	
**Visit**	**n (%)**	**n (%)**	**n (%)**	**n (%)**	**n (%)**	**Total**
Baseline	79 (75.2)	19 (18.1)	2 (1.9)	4 (3.8)	1 (1)	105
Day 14	71 (75.5)	19 (20.2)	3 (3.2)	1 (1.1)	0 (0)	94
Day 30	80 (84.2)	11 (11.6)	3 (3.2)	1 (1.1)	0 (0)	95

Global Assessment of Change

Global Assessment of Change was requested at each visit asking subjects to compare their symptoms prior to the start of the study up to the time of the visit. The subject was asked "How would you rate the change in eye dryness compared with immediately before your treatment?" A rating of +4 is complete or 100% improvement and -4 is 100% worsening.

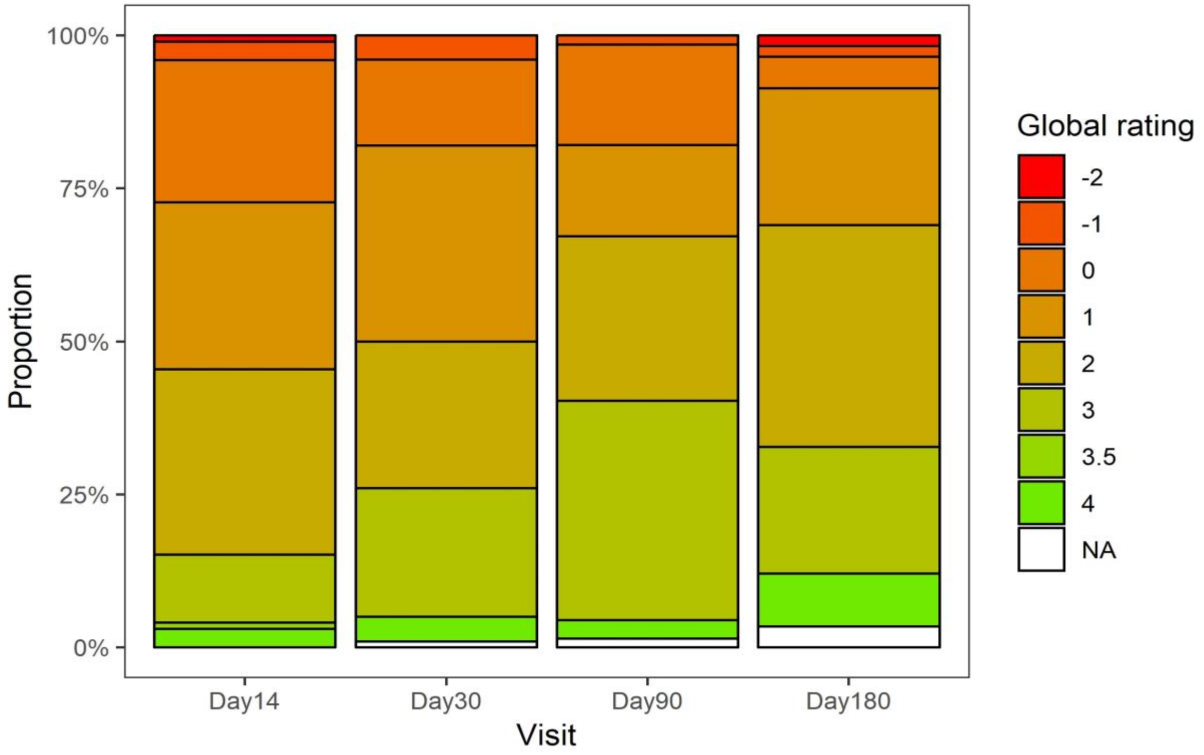

Figure S18: Global Assessment of Change

Usability

The subject was instructed to read the instructions for use and was also briefly trained by the physician. After use, the subject was given a survey regarding ease of use. The eye care provider also filled out a survey as to their perception of the ease of use and learning.

Table S19: User Usability

Rating	1	2	3	4	5	NA	All
Are the instructions easy to understand?	0	0	6	25	80	9	120
Button easy to press and hold down?	0	3	16	30	71	0	120
Device comfortable to hold?	0	2	9	24	85	0	120
Device easy to apply?	0	0	9	40	70	1	120
Device easy to charge?	0	0	10	16	81	13	120
Device easy to remove from packaging?	1	3	4	18	82	12	120
Instructions provide adequate information?	0	0	6	28	77	9	120

Table S20: Investigator assessment of subject’s ability to use the device

	No	Yes	N/A
Did the user find the device easy to remove from pkg	1 (0.8%)	109 (90.8%)	10 (8.3%)
Was the user able to hold the device and locate off/on switch?		116 (96.7%)	4 (3.3%)
Was user easily able to turn off/on	1 (0.8%)	115 (95.8%)	4 (3.3%)
Was user able to apply to the ext. nasal nerve and hold for 30 sec	2 (1.7%)	114 (95%)	4 (3.3%)
Was user able to determine available charge?		112 (93.3%)	8 (6.7%)
Was user able to determine how to charge the device?		115 (95.8%)	5 (4.2%)

Satisfaction

Subjects were asked to score their satisfaction with the device. Most subjects were satisfied or very satisfied with the device. Satisfaction tended to increase over time as the subjects learn how to use the device and apply it to their unique anatomy. There is also the possibility of some selection bias in which subjects who are more satisfied tend to remain in the study.

Table S21: Satisfaction

	Very satisfied	Somewhat satisfied	Neither satisfied nor dissatisfied	Dissatisfied	Very dissatisfied	Total
Visit	n (%)	n (%)	n (%)	n (%)	n (%)	Total
Day 14	35 (34.3)	45 (44.1)	20 (19.6)	0 (0)	2 (2)	102
Day 30	41 (40.6)	40 (39.6)	16 (15.8)	4 (4)	0 (0)	101
Day 90	29 (43.3)	29 (43.3)	6 (9)	3 (4.5)	0 (0)	67
Day 180	26 (44.8)	25 (43.1)	5 (8.6)	1 (1.7)	1 (1.7)	58

Recommend to friend

Subjects were asked whether they would recommend the device to a friend or family member. Table 37 shows that most subjects would recommend the device to a friend.

Table S22: Recommend to friend

	No	Not yet	Possibly	Maybe	Yes	
**Visit**	**n (%)**	**n (%)**	**n (%)**	**n (%)**	**n (%)**	**Total**
Day14	8 (8.2)	1 (1)	0 (0)	1 (1)	88 (89.8)	98
Day30	8 (8.2)	0 (0)	1 (1)	1 (1)	88 (89.8)	98
Day90	4 (6.1)	0 (0)	0 (0)	0 (0)	62 (93.9)	66
Day180	5 (8.8)	0 (0)	0 (0)	0 (0)	52 (91.2)	57

Use Again

Subjects were asked whether they would use the device again themselves. Most patients (about 90%) would use the device again.

Table S23: Use again

	No	Yes	
**Visit**	**n (%)**	**n (%)**	**All**
Day14	8 (7.9)	93 (92.1)	101
Day30	10 (10.1)	89 (89.9)	99
Day90	4 (6)	63 (94)	67
Day180	8 (13.8)	50 (86.2)	58

Compliance

Compliance was assessed in different ways. The subjects were asked to maintain diaries for both concomitant therapy and device usage. The most reliable method was the logs on the device in which the activation, the date, and the on time were recorded and downloaded when the subjects returned the device at the study exit. Several subjects who were early terminations between 31 and 180 days in fact continued to use the device. Several of these early terminations between 31 and 180 days were due to the subject not wanting to bring the device back for return.

Table S24: Usage

	n	Mean (SD)	Range
Duration of use, day 0-30, sec	93	26.9 (9.2)	[10 - 64]
Duration of use, day 31-180, sec	59	29.6 (11.3)	[8 - 71]
Number of uses, day 0-30	97	123.3 (79.6)	[7 - 513]
Number of uses, day 31-180	64	221.9 (239.6)	[0 - 1183]

Table S22: Number of uses

	Day 0-30	Day 31-180
0-50	13	16
50-150	59	17
150-250	18	10
More than 250	7	21
All	97	64

Concomitant Medication Usage

Subjects were asked to maintain stable regimens of ophthalmic medications during the 30-day time frame, and thereafter during 31-180 days they could make some changes. As far as systemic medications, investigators and coordinators were asked to identify subjects who were taking stable regimens of systemic medications to be enrolled in the study. Based on the questions posed by investigator at follow up visits, there were a total of 11 ophthalmic treatment changes in nine subjects. Four of these changes in four subjects was to decrease or eliminate a medication. One subject increased artificial tears at day 14 and then made multiple changes after 30 days but the logger for this subject showed that usage of the device stopped. One subject added artificial tears at day 90.

When the diaries were reviewed, it was found that a greater number of subjects had made changes to their medication regimens, artificial tears in particular. Thirty-four of the total number of subjects using artificial tears at baseline, 34/80 (43%) decreased artificial tear usage and 18/80 (23%) stopped artificial tear usage completely at 30 days.

There were very few changes to systemic medications. Most of the changes involved short term antibiotic regimens or other medications such as for diabetes which would not be expected to affect dry eye. One subject had a flair of rheumatoid arthritis and the infliximab and cortisone might affect dry eye therapy. These were added after the Day 30 visit. Two subjects added oral antihistamines which could also affect symptoms of dry eye if they are allergy related.

Adverse Events

A total of fifteen (15) ophthalmic Adverse Events (AEs) were recorded. No Serious Adverse Events (SAEs) related to the device occurred throughout the study. Six (6) were possibly related to the device and among them five were mild and one moderate. The most common adverse event was dizziness and lightheadedness occurring at a rate of 2-3%.

Table S25: Overall Adverse Events

Event Description	Day to AE	Duration (days)	Severity	Impact of Device	Action Taken
Inflammation due to Schirmer strip	13	14	Mild	No change	Drug Rx
Blurred vision	1	232*	Mild	No Change	New Contact Lenses
Eye Floater	92	1	Mild	No Change	None
Corneal Medicamentosa	91	60	Mild	No change	Decrease artificial tear usage
Corneal Neovascularization	180	21	Moderate	No change	None
Dry Eye Pain	2	19	Mild	No change	Artificial Tears
Eyelid irritation due to Schirmer strip	0	1	Mild	No change	None
Stye on lower lid OD	1		Mild	No change	Drug Rx
Vitreous Hemorrhage OS	64	16	Mild	Discontinued	None
Severe itching and sensitive eyes papillae OU	5	8	Moderate	Discontinued	Drug Rx
Eye infection OU	55	7	Mild	No change	None
Eye infection OU	74	8	Mild	No change	None
Eye infection OU	95	0	Mild	No change	None
Corneal Abrasion due to Schirmer strip		23	Moderate	No change	Drug Rx
Sjogren's syndrome	30	Ongoing	Mild	No change	Referred to rheumatologist

Two adverse (2) events were definitely related and both were of mild severity.

Table S26: Adverse Events definitely related to the device

Description	Day to AE	Duration (days)	Severity	Impact of Device	Action Taken
Slight headache, sneezing, tickling sensation	1	175	Mild	No change	None
Intermittent nose soreness	85	6	Mild	No change	None

Seven (7) adverse events were possibly related to the device.

Table S27: Adverse Events possibly related to the device

Description	Day to AE	Duration (days)	Severity	Impact of Device	Action Taken
Lightheadedness, dizziness	1	1	Mild	No change	none
Nausea, headache, lightheadedness, and dizziness after one treatment	0	30	Serious	Discontinued	Neurology referral
Dry eye pain	2	19	Mild	No change	Artificial Tears
Stye lower lid OD	1	22	Mild	No change	Drug Rx
Temporal Pain R	10	1	Mild	Interrupted	Drug Rx
Severe Itching and Sensitive Eyes Papillae OU	5	8	Moderate	Discontinued	Drug Rx
Headache	1	1	Mild	No change	None

* One adverse event, nausea, headache, lightheadedness, and dizziness was classified as a severe unanticipated adverse event. This subject used the device one time at the baseline visit and complained of ongoing symptoms afterward. She described them as continuous, but the symptoms did not affect her daily life activities. She was exited from the study and referred. She did have a neurologic evaluation and sought acupuncture, after which she improved.
